# Effectiveness of PRECEDE model for health education on changes and level of control of HbA1c, blood pressure, lipids, and body mass index in patients with type 2 diabetes mellitus

**DOI:** 10.1186/1471-2458-11-267

**Published:** 2011-04-28

**Authors:** Miguel A Salinero-Fort, Enrique Carrillo-de Santa Pau, Francisco J Arrieta-Blanco, Juan C Abanades-Herranz, Carmen Martín-Madrazo, Berta Rodés-Soldevila, Carmen de Burgos-Lunar

**Affiliations:** 1 Fundación Investigación Biomédica. Hospital Carlos III. SERMAS. Madrid. Spain; 2 Unidad de Nutrición. Hospital Ramón y Cajal. SERMAS. Madrid. Spain; 3 Unidad de Formación e Investigación. Área 4 de Atención Primaria. Madrid. Spain; 4 Unidad de Epidemiología Clínica. Hospital Carlos III. SERMAS. Madrid. Spain

## Abstract

**Background:**

Individual health education is considered to be essential in the overall care of patients with type 2 diabetes (DM2), although there is some uncertainty regarding its metabolic control benefits. There have been very few randomized studies on the effects of individual education on normal care in DM2 patients with a control group, and none of these have assessed the long-term results. Therefore, this study aims to use this design to assess the effectiveness of the PRECEDE (Predisposing, Reinforcing, Enabling, Causes in Educational Diagnosis, and Evaluation) education model in the metabolic control and the reduction of cardiovascular risk factors, in patients with type 2 diabetes.

**Methods:**

An open community effectiveness study was carried out in 8 urban community health centers in the North-East Madrid Urban Area (Spain). Six hundred patients with DM2 were randomized in two groups: PRECEDE or conventional model for health promotion education. The main outcome measures were glycated hemoglobin A1c, body mass index (BMI), blood pressure, lipids and control criteria during the 2-year follow-up period.

**Results:**

Glycated hemoglobin A1c and systolic blood pressure (SBP) levels decreased significantly in the PRECEDE group (multivariate analysis of covariance, with baseline glycated hemoglobin A1c, SBP, and variables showing statistically significant differences between groups at baseline visits). The decrease levels in diastolic blood pressure (DBP), triglycerides and LDL cholesterol were nonsignificant. PRECEDE increased compliance in all control criteria, except for LDL cholesterol. BMI did not change during the study in either of the two models analyzed.

**Conclusions:**

PRECEDE health education model is a useful method in the overall treatment in patients with type 2 diabetes, which contributes to decrease glycated hemoglobin A1c and SBP levels and increase the compliance in all the control criteria, except for LDL cholesterol.

**Trial registration number:**

ClinicalTrials.gov NCT01316367

## Background

Type 2 diabetes mellitus (DM2) is one of the chronic diseases that have increased in prevalence and incidence rates in recent years [1], and some authors consider it as the epidemic of the 21st century [2]. It is also associated with premature morbidity and mortality [3,4] as well as with an increase in healthcare costs [5].

Individual health education is considered to be essential in the overall care of patients with DM2, although there is some uncertainty regarding its metabolic control benefits. The PRECEDE (Predisposing, Reinforcing, Enabling, Causes in Educational Diagnosis, and Evaluation) model developed by Green and Kreuter [6] is one of the different educational models that focus on factors influencing health-related behavior, based on the relationship between the health professional and the patient, and is particularly appropriate for application in chronic diseases.

The efficiency of the PRECEDE model has been proven in different studies in the health enviroment, such as improving care habits among asthmatic children and improving medication adherence in patients with a chronic disease [7,8]; however, it has rarely been used in DM2.

There have been very few long-term studies, with randomized controls, on the effects of individual education in normal care in DM2 [9]. Therefore, the aim of this study was to assess the effectiveness of the PRECEDE education model on the changes in HbA1c, blood pressure (BP), lipids, and body mass index (BMI) in patients with DM2 over the long-term (2 years).

## Methods

### Patients

**Figure 1:**
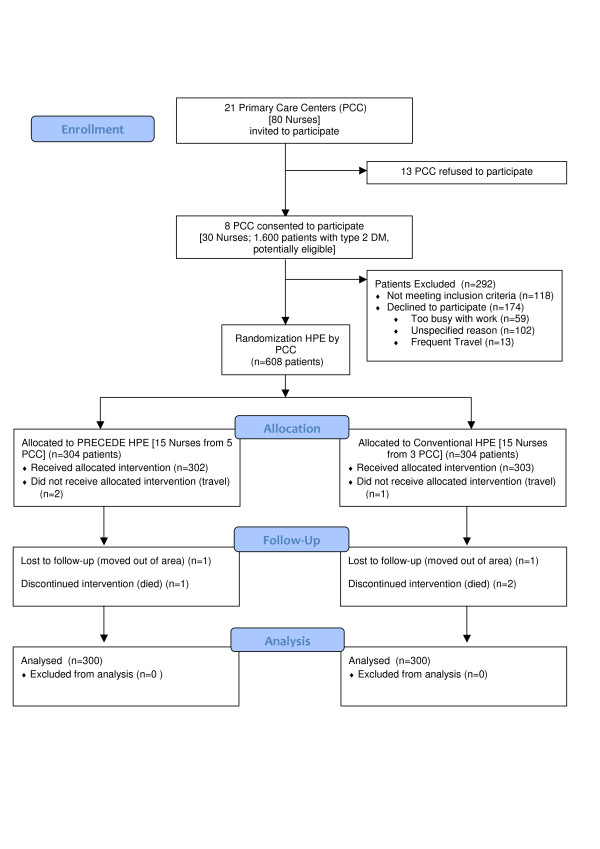
**Flow Diagram of participants**. HPE: Health Promotion Education.

We conducted an open community effectiveness study in which 21 Primary Care Centers (PCC), in the North-East Madrid Urban Area (Spain), were invited to participate; 13 refused, and 8 PCC were randomized in two arms: Precede Health Promotion Education (PHPE) and Conventional Health Promotion Education (CHPE). The participants at each PCC were randomized by selection from lists of patients with previously diagnosed DM2. Figure 1 shows the patient recruitment process. The research group was comprised of 33 persons: 30 nurses (15 in each group) and 3 scientific researches (technical group). A member of the clinical assistance team at each PCC was appointed as liaison officer between the PCC and the technical group. The study was approved by the ethics committee of the Hospital Ramón y Cajal.

Eligibility criteria for patients were: older than 30 years, with previously diagnosed DM2 (cardinal clinical, plus random blood glucose >200 mg/dl or oral glucose of >200 mg/dl at 2 h, twice, or plasma fasting glucose of >126 mg/dl on two occasions or being diagnosed previously, received specific treatment for DM2) and the exclusion criteria were: gestational diabetes, patients involved in clinical trials, patients with life expectancy less than 1 year (according to clinical judgment), patients who refused to participate, homebound patients. Patients meeting criteria for inclusion and not meeting any exclusion criteria were invited to participate, and were included after accepting and signing an informed consent form.

## Methods

Visits in both groups included the usual care and individual counseling, based on the CHPE or PHPE models, respectively.

The CHPE model was defined according to the recommendations of the Spanish Ministry of Health National Conference on Diabetes Mellitus [10], which was complemented by criteria for good care of the Madrid Primary Healthcare Service for the promotion of healthy lifestyles among adults (2004-2007). The model was based on the following aspects: self-monitoring of glycaemic control (patients were encouraged to monitor their blood glucose levels, to record these values and bring a record book to all subsequent appointments); physical exercise (this involved initiation of an exercise plan that could be incorporated into the patient's daily schedule, after taking into consideration their level of fitness, e.g. 1-h walk daily); diet (the patient was assisted with the identification of dietary behaviour that adversely influences blood glucose control, lipid levels, weight management, and times of the day when the patient was most vulnerable to overeating, and given improved understanding of the relative effects of certain food choices on blood glucose control); medication adherence; and smoking cessation (patients were encouraged to stop smoking by advising them about the danger of smoking to health, with emphasis on the increased dangers of smoking in diabetic patients).

The PRECEDE HPE model is a diagnostic tool behavior and therefore the first step in its implementation is to identify the behavior to be analyzed. The model considers the influence of the following three factors on health-related behavior:

-Predisposing: factors influencing the patient's motivation to undertake the behavior to be analyzed or encouraged.

-Facilitators: factors influencing the level of easiness or difficulty the patient and his/her family have in undertaking a given behavior.

-Reinforcing: factors arising after the patient has undertaken the behavior, and which reward or punish it.

Nurses in the experimental group had to answer the following question: What does the patient need to change behavior? (increasing physical activity, reducing the daily intake of bread, eat fewer times a day, medication adherence improvement, self-monitoring of blood glucose, improving skills for insulin treatment). After, two behaviors were selected for each patient. The nurses research-practitioners first looked at Predisposing factors that influence the analyzed behavior. Patient's responses and comments were written in two parallel lists: positive (+) and negative (-) factors in patients behaviors that need improvement. Predisposing factors are subjective (beliefs, opinions, values, thoughts, knowledge). Subsequently, factors that facilitate the studied behavior were analyzed. These are objective factors such as patient's skills or availability of resources. Finally, subjective Reinforcing factors (what the patient says after his/her behavior) and objectives (response to social and family environment, physical, emotional, and economic consequences).

Researchers in the experimental group received training in the PRECEDE model before patients were included in the study. This specific training involved two steps: first they were instructed about the basic, theoretical, and practical concepts involved in the application of the model. Second, they participated in a course on clinical interviews to improve their skills when dealing with patients.

Researchers in both groups were subsequently trained in the procedure to be used in the study in three sessions. These covered the criteria for including/excluding patients, collecting variables, collecting biochemical/biological parameters, resolving doubts, and piloting the data collection process with the first histories.

The study was carried out during a 2-year follow-up period (2003-2005) and the number of visits was identical for both groups: 10 visits (0 and 1 at month 1, were baseline visits; and 2 to 9, were follow-up visits, every 3 months).

**Figure 2:**
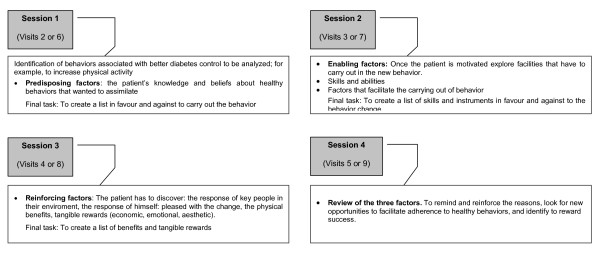
**Contents of the PRECEDE health promotion education model**.

Baseline data were collected during visits 0 and 1, and during visits 2 to 9 assigned models were applied and the data collected. The PRECEDE model application for each behavior analyzed took 4 visits (sessions), as shown in Figure 2. Nurses attended each patient with an average time of 40 minutes per session. Usual proceedings took an extra 20 minutes. Each nurse attended an average of 20 patients during the follow-up period.

The data gathered were sociodemographic variables (age, gender), hygienic and dietary habits, female menopause, tobacco consumption (cigarettes/day), alcohol consumption (alcohol units/week), physical activity practice (measured in hours per week considering any exercise or activity outside the regular job), self-monitoring of capillary glycemia, foot self-care, medication adherence (was measured using Haynes-Sackett test [11]: 'Most patients have difficulty taking tablets. Do you have any difficulty taking yours?' whose response was collected on a 5-point Likert scale **(**5, never; 4, seldom; 3, half of the time; 2, mostly; and 1, always. Values 4 and 5 were considered as medication adherence), associated morbidity (arterial hypertension, dyslipidemia, obesity, ischemic cardiopathies such as angina, acute myocardial infarction (AMI), and cerebrovascular accident (CVA), diabetes mellitus complications (microvascular, macrovascular, neuropathy), and the type of treatment prescribed (pharmacological and dietary). In follow-up visits data were collected on various biochemical-biological parameters (BMI, systolic blood pressure (SBP), and diastolic blood pressure (DBP), total cholesterol, high-density lipoprotein (HDL) cholesterol, low-density lipoprotein (LDL) cholesterol, triglycerides, and HbA1c). Blood pressure was measured according to the recommendations of the *Seventh Report of the Joint National Committee on Prevention, Detection, Evaluation, and Treatment of High Blood Pressure *(2003) [12].

The primary outcome was HbA1c, lipid levels, blood pressure, BMI after 24 months of follow-up.

### Sample size

For an alpha of 0.05, a power of 80%, and in order to detect an reduction of HbA1c of 0.3 percentage units, with a common standard deviation (SD) of 1.15, in the PRECEDE group, the overall sample size required was 462 patients (231 in each arm of the study). Since randomization was by PCC, the sample size had to be larger than if simple randomization had been performed, in order to consider the design effect (DE). The DE was calculated as follows: DE = 1 + (nc - 1) * ICC (where nc is the mean number of individuals in the cluster, and ICC the intracluster correlation coefficient). The ICC in the present work was deemed to be 0.01. The mean cluster size was assumed to be 30 patients. Given these assumptions, the final sample size required was 596 patients (298 in each arm).

### Statistical analysis

First, a descriptive analysis was carried out for each variable included in this study, involving the mean and SD for the quantitative variables and frequencies with confidence intervals of 95% (95% CI) for the qualitative variables. The Student's *t*-test or its nonparametric equivalent was used for paired data (Wilcoxon test). Furthermore, Pearson χ^2 ^test was used for the qualitative variables, and McNemar's test was used for paired data.

The change (mean end - mean start value) was calculated in both diabetological education models, for the following variables: total cholesterol, LDL cholesterol, HDL cholesterol, HbA1c, SBP, and DBP. The effect of the PRECEDE HPE was determined for these variables using the formula: mean value of the change in HPE PRECEDE - mean value of the change in conventional HPE. The covariance analysis methodology (ANCOVA) proposed by Vickers was used to determine the adjusted effect of PRECEDE [13]. The adjustment variables were: initial value and variables with statistically significant difference between groups at baseline visits (adherence to diet, adherence to medication, and type of treatment).

In all instances, the accepted level of significance was 0.05 or less, with 95% CI. All the analyses were carried out using the intention-to-treat principle. Statistical analysis of the data was carried out with SPSS 15.0 (SPSS, Inc., Chicago, Illinois).

## Results

A total of 608 patients were included, of which 51.6% were women, with a mean age of 66.7 years (SD: 14.5), and a natural history of disease mean of 9.1 years (SD: 8.3). A total of 304 patients were assigned to the conventional model and 304 to the PRECEDE model. The two groups studied according to the type of health promotion model were observed to be homogeneous in terms of gender, age, and DM2 evolution time. The baseline clinical characteristics of the two groups, the distribution of morbidity, and chronic complications are shown in Table 1.

**Table 1:** Baseline characteristics of participants by HPE Assignment.

	PRECEDE *n*=300	CONTROL *n*=300	*p *value
**Female **gender % (95% CI)	53.8 (50.6-57)	49.3 (46.1-52.5)	0.27
**Age **(years)	66.06 (8)	67.28 (19)	0.3
**DM duration **(years)	8.80 (8)	9.49 (8)	0.29
**Current Smoker **% (95% CI)	9.5 (6.3-12.7)	15.1 (4.6-25.6)	0.36
**No. of cigarettes/day **	11.88 (10)	12.36 (9)	0.84
**Alcohol/week **(units)	3.11 (8)	5.08 (30)	0.27
**Exercise **(hours/week)	7.55 (4)	7.98 (5)	0.25
**Self-control **% (95% CI)	67.9 (62.8-73)	64.2 (58.8-69.6)	0.33
**Self-management feet **% (95% CI)	65.7 (60.5-70.9)	93.6 (90.8-96.4)	<0.01
**Compliance with diet **% (95% CI)	55.7 (50.2-61.2)	74.2 (69.3-79.2)	<0.01
**Therapeutic compliance % **(95% CI) Always/Almost always	81.3 (77-85.6)	93.9 (91.2-96.6)	<0.01

**Medication profile **% (95% CI)			

Diet	79.5 (75.1-83.9)	72.8 (67.8-77.8)	0.05
Sulfonylureas	42.3 (36.9-47.7)	44.5 (38.9-50.1)	0.58
Meglitinide	4.1 (1.9-6.3)	1.3 (0-2.6)	0.03
Biguanides	25.6 (20.8-30.4)	19.7 (15.2-24.2)	0.08
Thiazolidinediones	0.3 (0.3-0.9)	1 (0-2.1)	0.29
Alpha glucosidase inhibitors	11 (7.6-14.4)	9 (5.8-12.2)	0.41
Insulin	14.2 (10.4-18)	14.4 (10.4-18.4)	0.94
Diuretics	27.4 (22.5-32.3)	23.1 (18.3-27.9)	0.23
Beta blockers	12.9 (9.2-16.6)	9.7 (6.4-13.1)	0.22
ACE inhibitors	30.6 (25.5-35.7)	29.1 (24-34.2)	0.68
ARB	14.5 (10.6-18.4)	12.4 (8.7-16.1)	0.47
Hypolipidemic	35.3 (30.1-40.6)	30.4 (25.2-35.6)	0.2
Calcium antagonist	15.1% (11.2-19)	19.1 (14.7-23.6)	0.19
Antiplatelet	22.4 (17.8-27)	17.7 (13.4-22)	0.15
Anticoagulants	3.2 (1.3-5.1)	3.7 (1.6-5.8)	0.74

**History of **% (95% CI)			

Hypertension	69.7 (64.6-74.7)	63.5 (58.1-69)	0.1
Dyslipidemia	48.7 (43.2-54.2)	44.8 (39.2-50.4)	0.33
CHD Angina	12.9 (9.2-16.6)	7 (4.1-9.9)	0.01
AMI	9.1 (5.9-12.3)	7 (4.1-9.9)	0.34
Stroke	3.2 (1.3-5.1)	6 (3.3-8.7)	0.1
Retinopathy	10.5 (7.1-13.9)	6.5 (3.7-9.3)	0.08
Nephropathy	5 (2.6-7.4)	4.1 (1.9-6.3)	0.6
Neuropathy	6 (3.4-8.6)	2.4 (0.7-4.1)	0.03

**Biochemical and biological parameters **			

Total Cholesterol (mg/dl)	203 (33)	205 (33)	0.45
HDL Cholesterol (mg/dl)	51 (14)	47 (11)	<0.01
LDL Cholesterol (mg/dl)	125 (29)	129 (28)	0.09
Triglycerides (mg/dl)	134 (70)	133 (76)	0.87
HbA1c (%)	7.05 (1.3)	7.36 (1.2)	<0.01
Body Mass Index (Kg/m^2^)	29.58 (4.58)	30.54 (5.16)	0.02
Systolic Blood Pressure (mmHg)	137 (165	134 (15)	0.02
Diastolic Blood Pressure (mmHg)	80 (8)	77 (8)	<0.01

**Values are given as mean (SD) unless otherwise specified**.

**CI: **Confidence Interval; **ACE**: Inhibitors of angiotensin converting enzyme; **ARB**: Inhibitors of the Renin Angiotensin II receptor; **CHD: **Coronary Heart Disease; **AMI**: Acute myocardial infarction.

The PRECEDE model led to a favorable variation in all parameters studied, while the conventional model group failed to achieve an improvement in HbA1c, triglycerides, or SBP, which showed a slight increase (Table 2).

**Table 2:** Mean values (SD) and changes of basal and final parameters in both groups.

	PRECEDE (***n*****: 300)**	CONTROL (***n*****: 300)**	Unadjusted PRECEDE effect (95% CI)	Adjusted PRECEDE effect (95% CI)
**Total Cholesterol (mg/dl)**				
Basal	203 (33)	205 (33)		
Final	194 (34)	194 (33)		
Change	-9.36 (33)	-10.88 (31)	1.51 (-3.6 to 6.6)	-0.10 (-4.8 to 4.56)
*p *value	0.67	0.88	0.56	0.97

**LDL Cholesterol (mg/dl) **				
Basal	125 (29)	129 (28)		
Final	118 (30)	122 (28)		
Change	-7.25 (30)	-7.27 (28)	0.02 (-4.6 to 4.7)	-2.64 (-6.9 to 1.6)
*p *value	0.1	0.11	0.99	0.22

**HDL Cholesterol (mg/dl) **				
Basal	51 (14)	47 (11)		
Final	52 (14)	51 (14)		
Change	0.8 (13)	3.7 (8)	-2.87 (-1.1 to -4.6)	-1.70 (-3.3 to -0.1)
*p *value	<0.01	0.51	<0.01	0.03

**HbA1c (%)**				
Basal	7.05 (1.3)	7.36 (1.2)		
Final	7.02 (1.2)	7.38 (1.1)		
Change	-0.03 (0.9)	0.04 (1)	-0.07 (0.2 to 0.1)	-0.18 (-0.3 to -0.04)
*p *value	<0.01	<0.01	0.40	0.01

**SBP (mmHg)**				
Basal	137 (15)	134 (15)		
Final	133 (13)	135 (16)		
Change	-4.22 (14)	0.18 (16)	-4.40 (-2 to -6.8)	-3.09 (-1.1 to -5.1)
*p *value	0.05	0.08	<0.01	<0.01

**DBP (mmHg)**				
Basal	80 (8)	77 (8)		
Final	77 (8)	76 (8.7)		
Change	-2.76 (8.9)	-0.75 (8.9)	-2.01 (-0.6 to 3.4)	-0.64 (-1.9 to 0.6)
*p *value	<0.01	0.27	0.40	0.32

**BMI (Kg/m2)**				
Basal	29,63 (4,50)	30,54 (5,16)		
Final	29,58 (4,58)	30,43 (5,19)		
Change	-0,05 (1,53)	-0,11 (1,58)	0,06 (0,30 a -0,19)	-0,03 (-0,29 a 0,24)
*p *value	0,56	0,24	0,64	0,85

**HbA1C**: Glycated hemoglobin; **SBP: **Systolic blood pressure; **DBP: **Diastolic blood pressure; **BMI**: Body Mass Index.

The non-adjusted effect of PRECEDE on the change in parameters was greater for HbA1c, triglycerides, DBP, and SBP, and was only significant in SBP. After adjusted analysis, the HbA1c levels decreased significantly (-0.18%; p = 0.01) in the PRECEDE model. Furthermore, SBP decreased by 3 mmHg (*p *< 0.01), and the decrease in DBP, triglycerides, and LDL cholesterol was nonsignificant. Furthermore, the total cholesterol remained unchanged (Table 2).

The BMI of the patients did not change during the study in either of the two models analyzed, and the adjusted effect of PRECEDE was close to zero (Table 2). In both models, the level of exercise decreased slightly and was not significant (5 min/week in the PRECEDE model and 22 min/week in the conventional model).

However, the PRECEDE model was better than the conventional model in percentage of subjects on-target for cardiovascular risk factors, after 2 years of follow-up: HbA1c <7% (*p *< 0.01), metabolic control (HbA1c <7% and LDL cholesterol <100 mg/dl) (*p *= 0.02), SBP <130 mmHg (*p *= 0.02), DBP <80 mmHg (*p = *0.01), BP control (<130/80 mmHg) (*p *< 0.01), and global control (metabolic and BP) (*p *= 0.02). Nevertheless, it was not significant for the criterion LDL <100 mg/dl and BMI <25 Kg/m^2 ^(Table 3).

**Table 3:** Percentage of Subjects On-Target for Cardiovascular Risk Factors at Baseline and at the End of the 24-Month Study Period, stratified by HPE.

Target	HPE	Baseline (%)	24 Months (%)	*p *value	Change (%)	*p *value
**HbA1C **(<7%)	Control PRECEDE	40.7 53.5	39 56	0.61 0.42	-1.7 +2.5	<0.01

**LDL **(<100 mg/dl)	Control PRECEDE	15.7 19.5	22 27	0.02 <0.01	+6.3 +7.5	0.55

**Metabolic control^1^**	Control PRECEDE	5.7 9.4	9 16.7	0.06 <0.01	+3.3 +7.1	0.02

**BMI **(<25 Kg/m^2^)	Control PRECEDE	12.7 12.3	12.5 12.3	0.90 1	-0.2 0	0.85

**SBP **(<130 mmHg)	Control PRECEDE	28.7 24.8	29.3 28	0.91 0.29	+0.6 +3.2	0.02

**DBP **(<80 mmHg)	Control PRECEDE	49 34	52.7 42.5	0.32 <0.01	+3.7 +8.5	0.01

**BP control^2^**	Control PRECEDE	21.3 15.4	21.7 18.9	1 0.21	+0.4 +3.3	<0.01

**Global Control^3^**	Control PRECEDE	0.7 1.9	1 4.4	1 0.06	+0.3 +2.5	0.02

1. HbA1c <7% and LDL cholesterol <100 mg/dl.

2. SBP <130 mmHg and DBP <80 mmHg.

3. Metabolic control and BP control.

**HPE: **Health Promotion Education; **SBP: **Systolic blood pressure; **BMI**: Body Mass Index; **DBP: **Diastolic blood pressure; **BP: **Blood pressure; **HbA1C**: Glycated hemoglobin.

## Discussion

There are currently very few studies on the efficacy of the PRECEDE model in patients with DM2. The study by Samaras et al [14], which aimed to increase physical exercise and the level of metabolic control in patients with DM2, observed an increase of 0.86% in HbA1c over 12 months in both the PRECEDE model and the conventional model, in contrast to the improvement in HbA1c levels observed in our study. The results of Samaras et al [14] might be owing to the limited intervention that lasted for 6 months and the PRECEDE group started with low levels of HbA1c (5.6%), leaving little room for improvement.

The reduction in HbA1c levels observed in our study is similar to that achieved by other health education strategies. The systematic review carried out by Duke et al [9] on the efficiency of individual health education in patients with DM2 showed a mean reduction in HbA1c of -0.23% after 6-9 months and -0.08% between 12 and 18 months.

Furthermore, the meta-analysis by Norris et al [15], which included eight clinical trials of self-management education for adults with DM2, showed a decreased HbA1c from baseline of -0.26% (95% CI -0,73 to +0.21%) at 1-3 months follow up, and of -0.26% (95% CI -0.05 to -0.48) at ≥ 4 months.

Finally, the DESMOND study [16] that assessed the effectiveness of a structured group educational program in patients recently diagnosed with DM2 obtained a nonsignificant adjusted result for the change in HbA1c of +0.05 after a follow-up period of 12 months, which is worse than that obtained in our study. In addition, the initial levels were worse than ours and the patients were "naive" in terms of health education.

In different pharmacological intervention studies, a decrease in HbA1c levels has shown a reduction in microvascular [17,18] and macrovascular [18] complications after long-term follow-up. These results as well as those obtained in our study suggest that pharmacological treatments need to be complemented with lifestyle-modifying strategies, such as the one proposed in the PRECEDE model.

The reduction in SBP obtained is observed to be greater than that found in studies carried out by Hiss et al [19], Ko et al [20], and Shibayama et al [21], which were included in the meta-analysis carried out by Duke et al [9]. In the latter study, the mean adjusted reduction, when compared with the usual management, was 1.86 mmHg, 12-18 months after the beginning of individual education. The decrease in our SBP levels is found to be relevant, as highlighted in the meta-analysis of 61 prospective and observational studies involving a million adults, carried out by Lewington et al [22]. The study showed a reduction of 7% in the risk of mortality owing to cardiovascular disease, and 10% in the risk of mortality owing to ictus with every 2 mmHg decrease in SBP.

The slight decrease in the lipid profile of total cholesterol, LDL cholesterol, triglycerides, and the slight increase in HDL cholesterol are found to be consistent with the findings observed previously by Samaras et al [14] and Gary et al [23]. The latter, in which the PRECEDE model was used to promote self-management in Afro-American DM2 patients, measured the effectiveness of four healthcare interventions based on primary healthcare and community services.

The difficulty that we faced in reducing the BMI may be partially explained by the similarity in the time spent on physical exercise in both groups, which did not improve during the follow-up.

Furthermore, a difficulty in reducing the BMI was mentioned in the meta-analysis by Boulé et al [24] and in other studies [15]. Finally, the work carried out by Scain et al [25], based on a group educational program focusing on self-management, also showed no differences when compared with normal care, although the BMI did decrease significantly when compared with the baseline.

We found no randomized studies with a control group, which evaluated the effect of educational models on metabolic control objectives (HbA1c <7% and LDL cholesterol <100), BP control (SBP <130 and DBP <80), or overall control (metabolic and BP <130/80 mmHg), indicating that our results cannot be compared.

However, on comparing the increase in the proportion of patients with metabolic control in the PRECEDE model obtained in our study with those of transversal studies carried out in primary healthcare, such as the one by Spann et al [26], we were able to find values that are similar, but lower than those found by Jackson et al [27] in a transversal study of 80,207 diabetic American veterans, of whom 38.9% achieved metabolic control, which can be partly explained by LDL levels (LDL = 109 mg/dl) that are substantially lower than those observed in our study.

The increase obtained in the proportion of patients with metabolic, blood pressure, and overall control after the application of PRECEDE model is relevant and suggests that there is a need to complement pharmacological treatments with lifestyle modification strategies like the one proposed by the PRECEDE model.

The most important limitation of this work is the nature of non-blind experimental studies, with the possibility of bias during response measurement, as researchers know which patients are members of the experimental group. Although, this bias is improbable because measurement of responses was objective, as it was based on results of analytical determinations. However, we believe that there could have been a possible Hawthorne bias effect, with a change in the behavior of the subjects owing to the knowledge that they are being studied. This would have had the same effect on both the groups, because the follow-up of the patients was stricter than normal in the two HPE arms, and they were all well aware of their participation in an experimental study when they signed the consent. A virtue of the study was that there were few losses, indicating that there was no selection bias owing to selective losses and that the analyses were carried out according to the intention-to-treat principle.

## Conclusions

As a result of all the above-mentioned factors, it can be concluded that the PRECEDE health promotion model is a useful method in the overall treatment of DM2 patients, because it contributes to significant decrease in HbA1c and SBP levels, as well as helps in increasing the compliance with all the control criteria, except for LDL cholesterol. Our findings indicate that further studies are necessary to substantiate these benefits. If they are confirmed, then the impact of the PRECEDE model should be evaluated in terms of cardiovascular morbimortality.

## Competing interests

The authors declare that they have no competing interests.

## Authors' contributions

MASF conceived of the study and participated in its design and perfomed the statistical analysis and drafted the manuscript. FJAB, JCAH, CBL drafted the manuscript and made substantial contributions to the analysis and interpretation. CMM participated in the design and coordinated the research group. ECSP, BRS helped in the statistical analysis and drafted the manuscript. All authors read and approved the final manuscript.

## Pre-publication history

The pre-publication history for this paper can be accessed here:

http://www.biomedcentral.com/1471-2458/11/267/prepub
